# Current trend of worsening prognosis of prostate small cell carcinoma: A population‐based study

**DOI:** 10.1002/cam4.2551

**Published:** 2019-09-13

**Authors:** Jili Wang, Xiaoyan Liu, Yan Wang, Guoping Ren

**Affiliations:** ^1^ Department of Pathology The First Affiliated Hospital Zhejiang University School of Medicine Hangzhou China; ^2^ Department of Pathology and Pathophysiology Zhejiang University School of Medicine Hangzhou China

**Keywords:** chemotherapy, malignant, prostate, small cell carcinoma, worse survival

## Abstract

**Objective:**

To determine the accurate age‐adjusted incidence of prostate small cell carcinoma (SCC), update the clinical and pathological characteristics, as well as survival data of prostate SCC from Surveillance, Epidemiology, and End Results (SEER) datasets.

**Methods:**

A total of 260 patients with prostate SCC were selected from the SEER database of the National Cancer Institute between 2004 and 2015. Age‐adjusted incidence (AAI) rates, the observed and relative survival rates were evaluated over time by the SEER*Stat Software version 8.3.5. Overall survival (OS) rates that stratified by summary stage and treatment effects were evaluated by Kaplan‐Meier method. The significant differences were assessed in a log‐rank test. Univariate and multivariate cox hazard regression analysis were performed to determine independent predictors of OS.

**Results:**

The incidence of prostate SCC has increased over time. The average age of prostate SCC patients was 70.25 years. More than 90% of tumors were poorly differentiated or undifferentiated. The majority of prostate SCC (77.7%) was at stage IV. 49% of patients had lymph node metastases and 68% of patients presented distant metastases (Compared with 60.5% of patients with distant metastases between 1973‐2003). Interestingly only 23.5% patients had high level PSA (>10 ng/mL). 58.8% of patients underwent chemotherapy, 25.4% of patients were treated by surgery, and 31.9% of patients were treated by radiotherapy. The observed survival rates of 1‐year, 2‐year, and 5‐year were 42.1%, 22.1%, and 12.5%, respectively (Compared with 47.9%, 27.5%, and 14.3%, respectively, between 1973 and 2003). Chemotherapy prolonged the OS of patients with regional (distant) metastases from 3 months (2 months) to 12 months (9 months). Multivariate cox regression analysis showed age, race, and stage were independent prognostic factors for prostate SCC patients.

**Conclusion:**

Prostate SCC is a highly malignant cancer and our analysis of recent data has shown its incidence is increasing. Incidence rate of metastatic prostate SCC has increased and the survival rates have worsened in recent years. However, chemotherapy shows some survival benefit for prostate SCC patients with regional and distant metastasis over other treatment methods. Further work is needed to understand the reason prognosis of this type prostate cancer is worsening.

## Graphical abstract

The incidence of prostate SCC is increased over time. The distant metastasis rate is increased and the survival rate of 1‐year, 2‐year, and 5‐year was decreased over time. Chemotherapy elongated the overall survival of regional/distant metastasis patients.

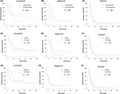

## 1 INTRODUCTION

Prostate small cell carcinoma (SCC) is a rare tumor, accounting for 0.5%‐2% of patients with prostate cancer.^1^ Previous studies have reported that prostate SCC patients lack specific clinical symptoms in the early stage. Once the symptomatic, including obstructive, neurological or systemic symptoms like paracancerous syndrome, bone pain, hydronephrosis, abdominal pain, bloody stools, tumor has usually already evolved to a terminal stage.^2^, ^3^, ^4^ Thus, the prognosis of patients diagnosed with prostate SCC is typically poor. Another reason for the poor prognosis is its aggressive behavior. This has been shown by numerous studies. Prostate SCC frequently leads to common osteolytic bone metastasis or visceral metastasis, rapid progression, and hormone therapy resistance.^1^, ^5^, ^6^, ^7^ In combination with these attributes of prostate SCC, no standard therapy has been established further exacerbating poor prognosis of the disease. The value of chemotherapy has been the subject of intense debate within this field. Papandreou et al conducted a study that showed doxorubicin failed to improve the survival of prostate SCC patients, whereas in a separate study, Moriyama et al showed four cycles of chemotherapy with combined cisplatin and etoposide achieved 3 years disease free survival.^8^ Similarly, there is also disagreement on the value of surgery and radiotherapy for prostate SCC.

Owing to the rarity of prostate SCC, studies usually stem from case reports or case series. The lack of a large population‐based study has been a limitation in this field for many years. Prior to 2011, a study based on SEER database that contains 191 prostate SCC samples had elucidated the clinical features and the prognosis of the disease, but the study lacked information on chemotherapy and failed to measure its value. Further study is needed to determine if the clinical characteristics and survival rates for prostate SCC have changed. Additionally, there is a relative paucity of large population‐based study investigating the real value of surgery, chemotherapy and radiation in the treatment of prostate SCC.

In the study, we analyzed recent data on prostate SCC from the SEER database (2004‐2015) with the four key aims. First, determine the accurate age‐estimated incidence of prostate SCC. Second, update the clinical characteristics and pathological features of prostate SCC. Third, update the survival rates and delineate the factors that affect the prognosis of SCC. Lastly, clarify the value of different treatments in the SCC.

## 2 MATERIALS AND METHODS

### 2.1 Study population

We used The Surveillance, Epidemiology, and End Results (SEER) database that consists of 18 population‐based tumor registries released in November 2017 for this analysis. The SEER program of the National Cancer Institute collects information (including demographic, tumor characteristics, among others) on approximately 28% of the United States population.

Prostate small cell carcinoma (SCC) was identified according to the ICD‐0‐3/WHO 2008 with the code: 8041/3 Small cell carcinoma, NOS. The eligibility of the criteria included: (a) tumors sequence number labeled “One primary only”; (b) the year of diagnosis ranging from 2004 to 2015; (c) with the information of survival months. Final cohort contains 260 prostate SCC patients that meet these criteria.

### 2.2 Definition of variables

Patients' demographic variables such as age at diagnosis, gender, race, and marital status were obtained. Tumor characteristics including tumor grade, SEER summary stage, American Joint Committee on cancer 6th edition (AJCC stage 6th), TNM stage, serum Prostate Specific Antigen (PSA), Gleason Score, etc, were extracted from the database. Furthermore, we collected treatment modality including surgery, radiation therapy, and chemotherapy information.

### 2.3 Statistical analyses

Incidence rates per 100 000 age‐adjusted to the population were collected by the SEER*Stat Software version 8.3.5 (Surveillance Research Program, National Cancer Institute, seer.cancer.gov/seerstat). The observed and relative survival rates were also calculated by the SEER*Stat Software version 8.3.5. Overall survival (OS) rates that stratified by summary stage and treatment effects were evaluated by Kaplan‐Meier method. The significant differences were assessed in log‐rank tests. Furthermore, univariate and multivariate cox proportional hazards models were performed to estimate the associations between covariates and prognosis. Only the significant variables from univariate analysis were enrolled in the multivariate analysis. Statistical analysis was performed with SPSS Statistical Package version 25.0 (SPSS Inc), and *P* < .05 was considered statistically significant.

## 3 RESULTS

### 3.1 The incidence of prostate SCC

**Figure 1:**
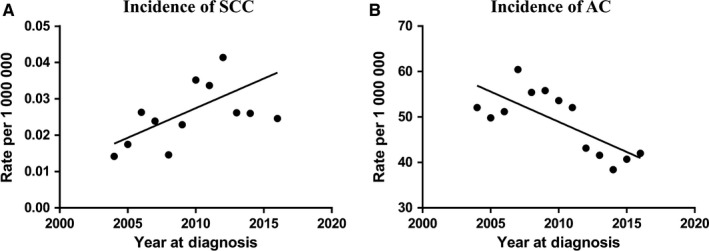
The incidence of prostate small cell carcinoma (SCC) and prostate adenocarcinoma (AC) during 2004‐2015

The overall age‐adjusted incidence (AAI) of prostate adenocarcinoma has decreased from 520.888 per 1 000 000 in 2004 to 407.088 per 1 000 000 in 2015. However, as shown in Figure 1, AAI of prostate SCC over time has increased from 0.142 per 1 000 000 in 2004 to 0.503 per 1 000 000 in 2015.

### 3.2 The clinical characteristics of prostate SCC

Table 1 provides an overview of the clinical characteristics of prostate SCC. Demographic characteristics showed the average age of prostate SCC patients was 70.25 years. The majority of patients were white (82.3%) and married (63.8%).

**Table 1:** Patient demographics and clinical characteristics (n = 260)

Characteristics	Level	Number (%)
Age at diagnosis	Mean ± SD	70.25 ± 11.343
	Median (range)	70 (30‐96)
	≤70	137 (52.7%)
	>70	123 (47.3%)
Race	White	214 (82.3%)
	Black	28 (10.8%)
	Others/Unknown	18 (6.9%)
Marital status	Married	166 (63.8%)
	Unmarried	77 (29.3%)
	Unknown	17 (6.5%)
Tumor grade	Moderately differentiated	7 (2.7%)
	Poorly differentiated	74 (28.5%)
	Undifferentiated	24 (9.2%)
	Unknown	155 (59.6%)
AJCC stage	II	27 (10.4%)
	III	4 (1.5%)
	IV	202 (77.7%)
	Unknown	27 (10.4%)
AJCC T stage	T1	31 (11.9%)
	T2	65 (25.0%)
	T3	32 (12.3%)
	T4	75 (28.8%)
	TX	57 (21.9%)
Lymph node metastases	N0	104 (40.0%)
	N1	103 (39.6%)
	NX	53 (20.4%)
Distant metastases	M0	72 (27.7%)
	M1	159 (61.2%)
	MX	29 (11.2%)
Summary stage	Localized	31 (11.9%)
	Regional	48 (18.5%)
	Distant	161 (61.9%)
	Unknown	20 (7.7%)
PSA	≤4	70 (26.9%)
	4‐10	42 (16.2%)
	10‐20	20 (7.7%)
	20‐50	19 (7.3%)
	>50	22 (8.5%)
	Unknown	87 (33.5%)
Surgery	Yes	66 (25.4%)
	None/Unknown	193 (74.2%)
Radiation therapy	Yes	86 (31.9%)
	None/Unknown	174 (66.9%)
Chemotherapy	Yes	153 (58.8%)
	No/Unknown	107 (41.2%)

Tumor characteristics showed data concurrent with the known aggressive behavior of prostate SCC. For cases with Gleason Score information (25), 72% had scores of 8‐10 (data not show). In cases with tumor grade information, more than 90% had poorly differentiated or undifferentiated tumors. Consistent with these data, 77.7% of patients were stage IV prostate SCC. As for T stage, T2 (25%) and T4 (28.8%) were the top two. Furthermore, regional invasion or distant metastases were common, with 49% lymph node metastases and 68% distant metastases. Overall, these results showed that prostate SCC has a high degree of malignancy with frequent and widespread metastasis. Surprisingly, the serum biomarker PSA, which is typically increased in aggressive prostate cancers, was normal for most prostate SCC patients, and only 23.5% of patients had high PSA levels (>10 ng/mL).

As for the treatment modalities used, chemotherapy was the primary (60%) therapy for SCC patients. 25.4% of patients were treated by surgery, and 31.9% of patients were treated with radiotherapy.

### 3.3 The prognosis of prostate SCC

To assess the survival rates of the patients diagnosed between 2004 and 2015, we calculated the observed and relative survival rates by the SEER*Stat Software version 8.3.5. The observed survival rate of 1‐year, 2‐year, and 5‐year were 42.1%, 22.1%, and 12.5%, respectively (Table2).

**Table 2:** Observed and relative survival rates of prostate SCC patients from 2004 to 2015

Years	Observed survival (SE)	Expected survival	Relative survival (SE)
1	42.1% (5.2%)	95.9%	43.9% (5.4%)
2	22.1% (4.5%)	92.5%	23.8% (4.9%)
3	13.8% (3.9%)	89.4%	15.2% (4.3%)
4	12.5% (3.7%)	86.7%	14.1% (4.3%)
5	12.5% (3.7%)	84.0%	14.1% (4.3%)

Note

Method is Kaplan‐Meier. Cumulative expected method is Ederer II. The observed and relative survival rates were also calculated by the SEER*Stat Software version 8.3.5.

**Figure 2:**
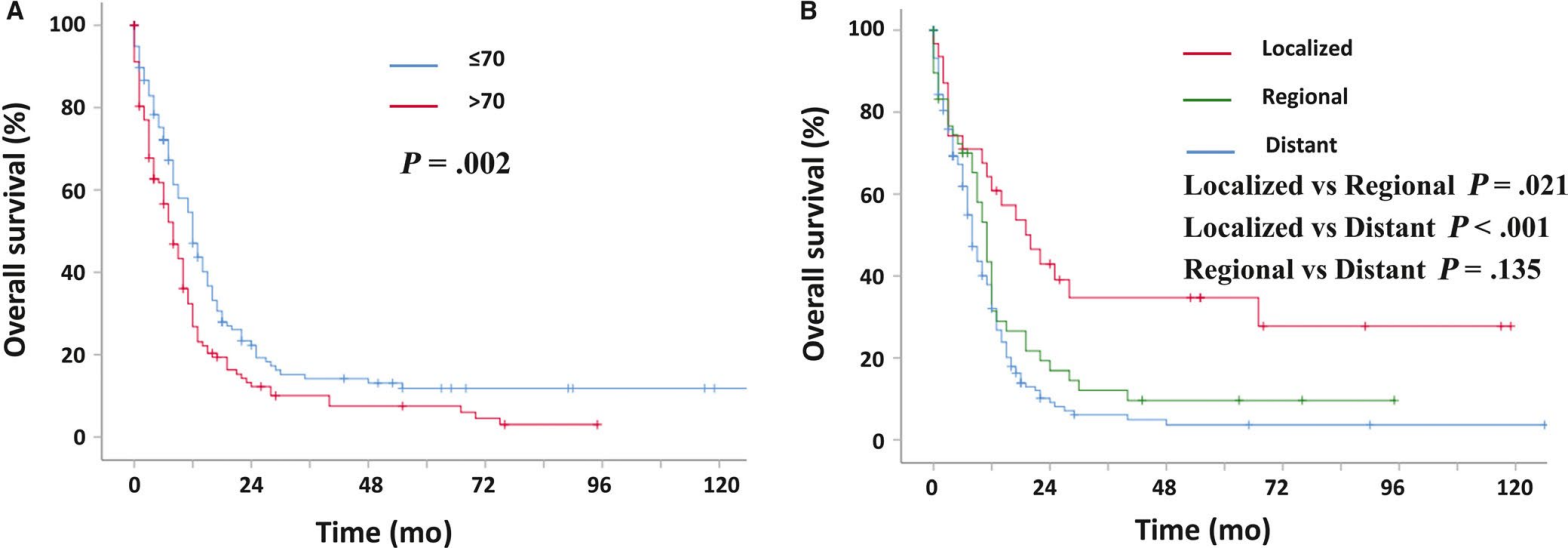
Kaplan‐Meier estimated survival curve of patients with prostate SCC patients. A: based on age at the time of diagnosis; B based on the summary stage (localized, regional, and distant)

As shown in Figure 2, the prognosis of young patients was better than that of elderly patients with median survival of 11 vs 8 months (*P* = .002). In addition, the survival was related to summary stage, with median survival of 20 months in localized tumor patients, 11 months in regional tumor patients, and 8 months in distant metastasis patients (localized vs regional *P* = .021; localized vs distant *P* < .001; regional vs distant *P* = .135).

**Figure 3:**
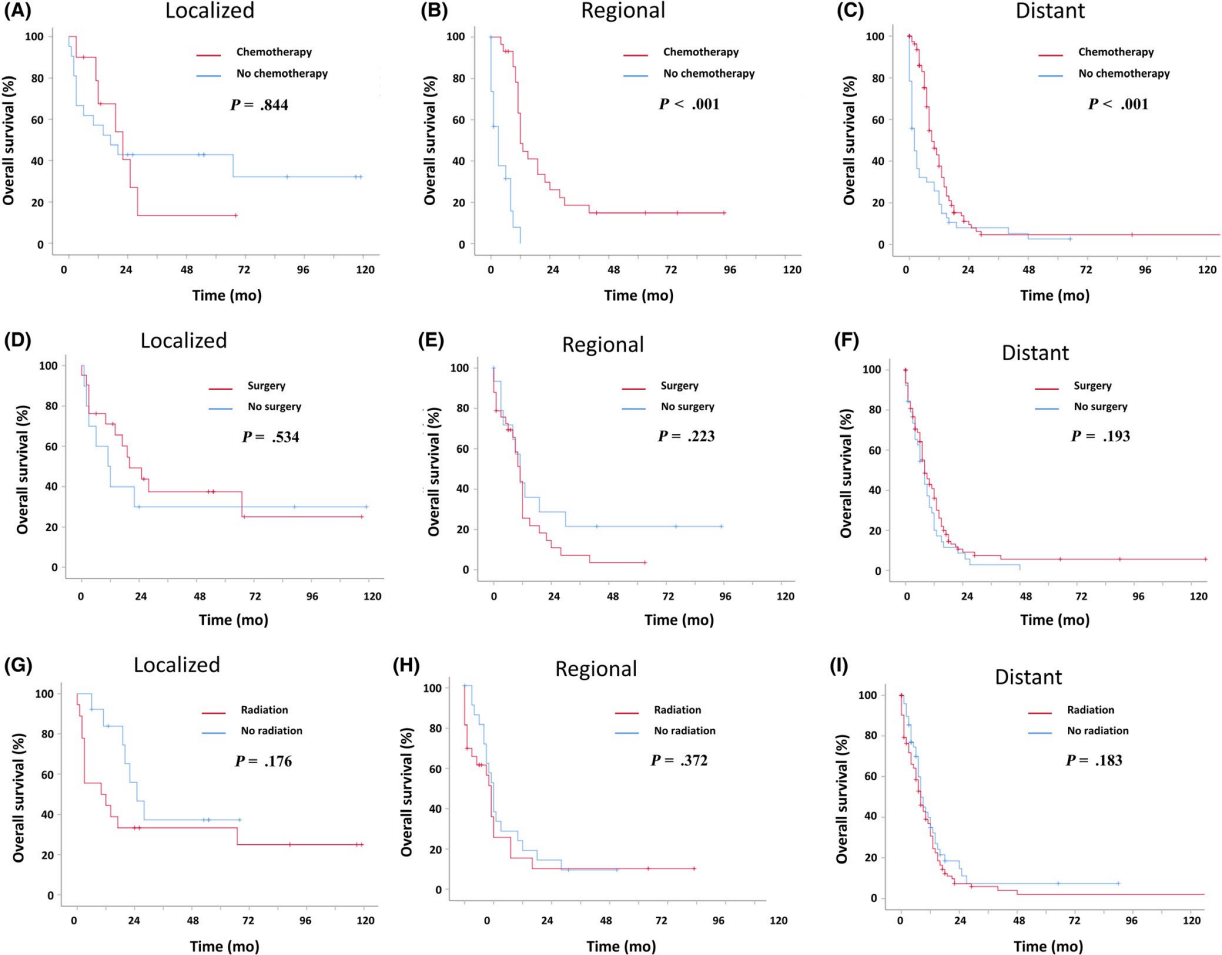
Kaplan‐Meier estimated survival curve of patients with prostate SCC patients based on treatment modality. (A‐C) based on chemotherapy. A: Localized prostate SCC patients; B: Regional prostate SCC patients; C: Distant prostate SCC patients. (D‐F) based on surgery. D: Localized prostate SCC patients; E: Regional prostate SCC patients; F: Distant prostate SCC patients. (G‐I) based on radiation. G: Localized prostate SCC patients; H: Regional prostate SCC patients; I: Distant prostate SCC patients

As for treatment methods stratified with summary stage, chemotherapy was effective for regional and distant metastases tumors, but not for localized tumors (Figure 3A‐C). Specifically, chemotherapy prolonged the median survival of distant metastases patients from 2 months to 9 months, increased the median survival of regional invasion patients from 3 months to 12 months. However, other treatments such as surgery and radiation showed no significant effect for prostate SCC when stratified by summary stage (Figure 3D‐I).

Univariate and multivariate cox regression results were shown in Table 3. Univariate cox regression analysis suggested that older age (HR: 1.495; 95%CI: 1.141‐1.959; *P* < .05), others/unknown race (HR: 1.837; 95%CI: 1.062‐3.179; *P* < .05), stage IV (HR: 2.747; 95%CI: 1.631‐4.625; *P* < .05), lymph node metastasis (HR: 1.578; 95%CI: 1.156‐2.155; *P* < .05), distant metastasis (HR:1.808; 95%CI: 1.302‐2.512; *P* < .05), no radiotherapy (HR: 1.353; 95%CI: 1.011‐1.811; *P* < .05) were risk factors for poor prognosis. Multivariate cox regression analysis showed age, race, and stage were independent prognostic factors for prostate SCC patients.

**Table 3:** Univariate and multivariate analyses for overall survival (OS) of patients

Variables	Level	Univariate		Multivariate	
		HR (95% CI)	*P* value	HR (95% CI)	*P* value
Age	≤70	1		1	
	>70	1.495 (1.141‐1.959)	.004	1.709 (1.271‐2.299)	<.001
Race	White	1		1	
	Black	1.176 (0.761‐1.817)	.465	1.770 (1.108‐2.826)	.017
	Others/Unknown	1.837 (1.062‐3.179)	.030	2.059 (1.162‐3.647)	.013
Marital status	Married	1			
	Unmarried	1.272 (0.939‐1.722)	.120		
	Unknown	0.985 (0.578‐1.680)	.857		
AJCC stage	II	1		1	
	III	0.668 (0.153‐2.924)	.593	0.474 (0.107‐2.108)	.327
	IV	2.747 (1.631‐4.625)	<.001	2.279 (1.205‐4.311)	.011
	Unknown	1.816 (0.964‐3.420)	.065	1.246 (0.517‐3.003)	.625
AJCC T stage	T1	1			
	T2	1.035 (0.634‐1.691)	.891		
	T3	1.123 (0.644‐1.960)	.682		
	T4	1.414 (0.880‐2.271)	.152		
	TX	1.257 (0.773‐2.045)	.356		
Lymph Node metastases	N0	1		1	
	N1	1.578 (1.156‐2.155)	.004	1.223 (0.871‐1.718)	.244
	NX	1.438 (1.002‐2.065)	.049	1.396 (0.870‐2.239)	.166
Distant metastases	M0	1		1	
	M1	1.808 (1.302‐2.512)	<.001	1.101 (0.732‐1.657)	.644
	MX	1.286 (0.802‐2.061)	.296	0.817 (0.394‐1.692)	.586
Surgery	Yes	1			
	No/unknown	0.945 (0.696‐1.284)	.718		
Radiation therapy	Yes	1		1	
	No/unknown	1.353 (1.011‐1.811)	.042	1.216 (0.883‐1.673)	.231
Chemotherapy	Yes	1			
	No/unknown	1.309 (0.992‐1.728)	.057		

Note

HR: Hazard ratio; Only variables that were significantly associated with survival in the univariate Cox analysis were included in the multivariate Cox analysis.

## 4 DISCUSSION

Many recent studies have shown that the overall incidence of prostate adenocarcinoma (AC) is decreasing,^9^, ^10^ however, very little was known for the specific incidence of prostate SCC. Our results showed the AAI of prostate SCC has been increasing in the United States. A possible explanation for the increased trend is the introduction of new highly potent androgen receptor‐targeted therapies (like abiraterone and enzalutamide). Data from several studies suggest that resistance developed from these therapies may be accompanied by the emergence of prostate SCC.^11^, ^12^ All in all, we should monitor and improve our understanding of this trend.

Prostate SCC is rare, however, aggressive malignancy with poor prognosis.^13^ Our study found that the majority of prostate SCC were poorly or undifferentiated and/or at high pathology grade. The major Gleason score was 8‐10, and nearly 70% of patients were accompanied by distant metastasis. In accordance with the present results, previous studies have demonstrated that prostate SCC was poorly differentiated, with high Gleason Score and widespread metastasis.^1^, ^2^, ^14^, ^15^, ^16^ What is surprising is that the distant metastasis rate from SEER database increased from 60.5% to 62% (reported by Deorah between 1973‐2003 and Wang J between 1973‐2004) to the 68% in this study (showed by our results between 2004 and 2015).^14^, ^17^ Additionally, we found that most patients showed normal serum PSA levels in contrast to other prostate carcinomas, and these results together with other studies showing prostate SCC patients without elevated PSA, may lead to missed diagnosis in these patients.^1^, ^5^, ^18^

Due to the aggressive behavior of SCC and delayed diagnosis at later stage, the median survival time for patients is short, with an average of 8 to 16 months.^6^, ^7^, ^18^ Our study found that the 1‐year, 2‐years, and 5‐years survival rates for prostate SCC patients were 42.1%, 22.1%, and 12.5%, respectively, lower than that previously reported (47.9%, 27.5%, and 14.3%, respectively).^14^ Additionally, the distant metastasis rate also showed a marked increase (68% vs 60.5%). One possible explanation for this phenomenon is that prostate SCC has become more malignant than previously recognized. It is important to bear in mind the possible bias in these results, thus, poorer prognosis and the increased rate of metastasis need to be interpreted with caution. For example, misdiagnosis may play a part in the survival rate, especially in early years. That is, when high‐grade adenocarcinoma was misdiagnosed as SCC due to the technology‐based restrictions, skewing survival upward. Because high‐grade adenocarcinoma responded well to androgen receptor‐targeted therapies, the survival rate was better in patients with high‐grade adenocarcinoma than patients with SCC. Thus, the survival of true prostate SCC might be overestimate.^14^ Currently, immunohistochemical (IHC) biomarkers including chromogranin, synaptophysin, CD56, insulinoma‐associated protein 1 (INSM1) has increased accuracy for SCC diagnosis,^19^, ^20^ which may aid in our understanding of survival rates as we monitor them going forward. However, our study should highlight the need to follow this trend and work to understand its reasons if it continues.

Due to its rarity, prostate SCC lacks standard treatment modalities. In clinical experiments, the treatment of prostate SCC is usually based on experiences with pulmonary small cell carcinoma.^1^ With respect to nonmetastasis prostate SCC, adjuvant chemotherapy with prostatectomy are recommended. With respect to metastasis prostate SCC, chemotherapy is regarded as the backbone; with other treatments such as androgen deprivation treatment (ADT), radiation, and AURKA inhibitors being debated.^1^, ^21^ For example, radiation is usually combined with chemotherapy, or serve as local palliative care for patients with severe obstructive symptoms or when there are no other treatment options available in the advanced stage.^22^ While hormone deprivation therapy is regarded as a treatment for prostate SCC mixed with adenocarcinoma.^21^

To date, previous studies based on SEER dataset lacked the information on chemotherapy and failed to measure the value of chemotherapy in treatment of prostate SCC.^14^, ^17^ In this study, we stratified the treatments based on summary stage and found that chemotherapy improved the survival of patients with distant/regional metastasis but not patients with localized tumor. These results agreed with the findings of other studies, in which they demonstrated chemotherapy is beneficial to patients with prostate SCC.^6^, ^18^

Furthermore, our results of multivariate cox regression analysis showed that age, race and stage were independent prognostic factors for prostate SCC patients. This finding is consistent with that of Deorah et al who reported that age, tumor grade, and stage were strong predictors of survival. We also found high tumor grade was associated with poor outcome in univariate cox regression analysis (data not show); however, 60% of patients lacked the information on tumor grade, thus, we did not include the tumor grade into Table 3.

Although the SEER database has allowed for an exploratory analysis of the rare prostate SCC, it is important to understand some limitations. First, the study has shortcomings by its retrospective nature. Second, the detail of which chemotherapy treatment used is not available in the public SEER database, which may restrict the research of the drug‐type‐specific effect. Prior studies have showed that doxorubicin failed to improve the prognosis, whereas platinum prolonged the survival months of patients with prostate SCC.^6^, ^7^ Thus, different drug type may be essential for providing the best treatments for prostate SCC. A further study with more focus on specific chemotherapy drugs and effect on patient prognosis and statistics is therefore suggested. Third, misclassification may be a concern of the analysis based on SEER database since there is no centralized review by a pathologist. Finally, the numbers of patient with prostate SCC are limited even though it is the most extensive database to date. One of drawbacks of the limited sample size in our study is that we cannot further analyze combined therapy. For instance, among the patients with localized prostate SCC, only 3 patients received surgery plus chemotherapy which is not enough data for survival analysis. In the future, it will be important to explore the potential use of combination‐based therapies on a larger population of prostate SCC.

## 5 CONCLUSION

Prostate SCC is a highly malignant cancer and its incidence is increasing in recent years. The metastasis incidence is also increased and the survival rate is worse than ever showing a trend toward poorer overall prognosis. We found chemotherapy has a survival benefit for prostate SCC with regional and distant metastasis. Further work is needed to establish the therapeutic efficiency of chemotherapy, surgery, and radiation on prostate SCC and more research is required to examine the negative trends observed in prostate SCC.

## CONFLICT OF INTEREST

The authors have declared that no conflict of interest exists.

## Data Availability

All raw data in this article can be obtained in the SEER program or by emailing the corresponding author.
